# Supplementary material for: The Effect of Different Factors on Poly(lactic-co-glycolic acid) Nanoparticle Properties and Drug Release Behaviors When Co-Loaded with Hydrophilic and Hydrophobic Drugs
Source: Polymers (Basel). 2024 Mar 22;16(7):865. doi: 10.3390/polym16070865 (PMC11013797; doi:10.3390/polym16070865)
Supplement: Supplementary file 1 [file polymers-16-00865-s001.zip › polymers-2864677-supplementary.pdf]

The Effect of Different Factors on Poly(lactic-co-glycolic acid) Nanoparticle Properties and Drug Release Behaviors When Co-Loaded with Hydrophilic and Hydrophobic Drugs

Lianguo Wang 1,2,3,†, Pei Wang 1,2,3,*,†, Yifan Liu 1,2,3, Muhammad Atae Mustafa Mahayyudin 1,2,3, Rong Li 1,2,3, Weilun Zhang 1,2,3, Yilan Zhan 1,2,3 and Zhihua Li 1,2,3

1 School of Stomatology, Jiangxi Medical College, Nanchang University, Nanchang 330006, China; guoguo19990713@163.com (L.W.); lyf070626@163.com (Y.L.); 413009220060@email.ncu.edu.cn (M.A.M.M.); lirong012@email.ncu.edu.cn (R.L.); 4207122044@email.ncu.edu.cn (W.Z.); 4207120011@email.ncu.edu.cn (Y.Z.); lwlq323@163.com (Z.L.)

[2] *Jiangxi Province Key Laboratory of Oral Biomedicine, Nanchang 330006, China*

[3] *Jiangxi Province Clinical Research Center for Oral Diseases, Nanchang 330006, China*

***Correspondence:** ndfskqyy620@ncu.edu.cn

† These authors contributed equally to this work.

Supplementary Material

1 Supplementary Figures

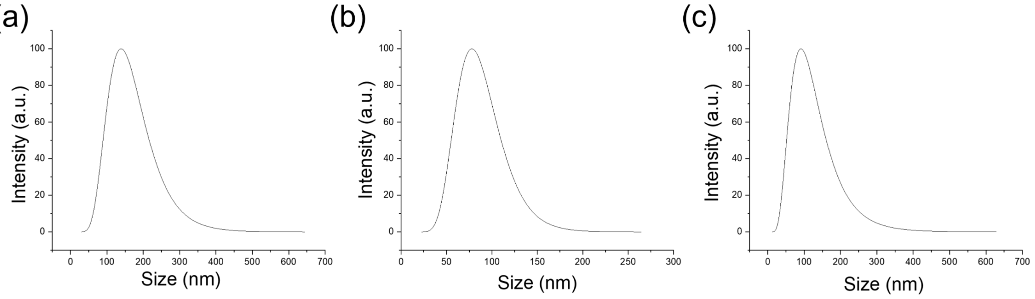

Supplementary Figure S1. Size distribution of PLGA NPs prepared in different organic phases (a) DCM, (b) EA and (c) PC under 2% BSA concentration and 4 mL water phase.

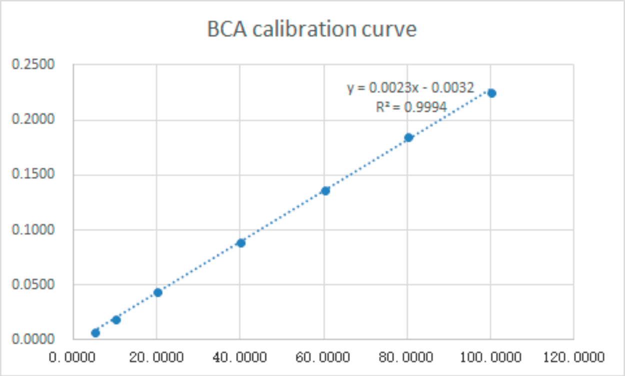

Supplementary Figure S2. Standard curve of BSA

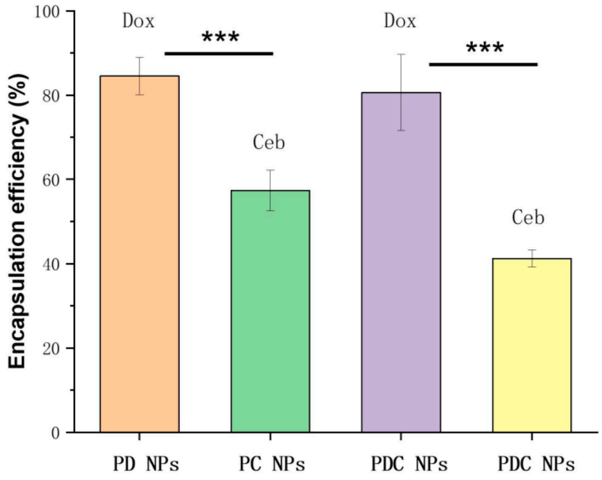

Supplementary Figure S3. The encapsulation efficiency of PD NPs, PC NPs and PDC NPs. ***$P < 0.001$.
